# Supplementary material for: ntSynt: multi-genome synteny detection using minimizer graph mappings
Source: BMC Biol. 2025 Dec 29;23:367. doi: 10.1186/s12915-025-02455-w (PMC12752294; doi:10.1186/s12915-025-02455-w)
Supplement: Supplementary file 1 — Additional file 1: Fig. S1–S16. Table S1–S18. [file 12915_2025_2455_MOESM1_ESM.pdf]

Additional file 1 for: "ntSynt: multi-genome synteny detection using minimizer graph mappings"

Lauren Coombe, Parham Kazemi, Johnathan Wong, Inanc Birol, René L. Warren

Table of Contents

Fig. S1: ntSynt pipeline overview. 3

Fig. S2: ntSynt common Bloom filter construction using a cascading approach. 4

Fig. S3: Simplifying the ntSynt minimizer graph. 5

Fig. S4: Converting linear graph paths to synteny block coordinates. 6

Fig. S5: Indel detection in ntSynt. 6

Fig. S6: Extension of ntSynt synteny blocks using minimizers computed with lower window sizes. 7

Fig. S7: Merging collinear synteny blocks. 8

Fig. S8: **Evaluating the correctness (or accuracy) of syntenic sequence blocks on controlled (synthetic) data.** 8

Fig. S9: **Simulated rearrangement size distribution.** 9

Fig. S10: Synteny block contiguity versus accuracy across increasing divergence levels in simulated human genomes. 10

Fig. S11: **Synteny accuracy across different minimum block length thresholds at various simulated sequence divergence rates (0.1-5.5%, facet plots).** 11

Fig. S12: Tool-relative error rates by simulated structural variant (SV) type across sequence divergence levels (0.1-5.5%, facet plots) in the controlled experiment. 12

Fig. S13: Pairwise dot plots between human chromosome 11 and the chromosome 11 sequence of three other primate genome assemblies: bonobo, chimpanzee and gorilla. 13

Fig. S14: IGV Screenshot showing alignments of gorilla long reads to the human reference genome. 14

Fig. S15: *Andrena* phylogenetic tree. 15

Fig. S16: Average synteny coverage of ntSynt blocks between human, mouse and rat reference genomes, sweeping on the *k*-mer size. 16

Table S1: Output format of ntSynt synteny blocks file. 17

Table S2: Default ntSynt parameter settings based on the user-supplied divergence (--*divergence*). 17

Table S3: Parameter settings used when running synteny block comparator tools. 17

Table S4: Reference genome assemblies used for synteny block analysis with ntSynt, SibeliaZ and SyntenyPortal. 18

Table S5: Mapped read statistics from aligning gorilla PacBio HiFi reads (individual Kamilah) to the gorilla reference genome build used in the synteny tests (Kamilah_GGO_v0) and a newer gorilla reference genome build (mGorGor1). 18

Table S6: Genome sequence assemblies used for synteny block analysis with ntSynt and SibeliaZ. 18

Table S7: Genome assemblies from the genus *Andrena* analyzed using ntSynt. 19

Table S8: Expected synteny block statistics for the pairwise comparisons between the human reference genome (T2T build) and one SURVIVOR [3]-simulated rearranged genome sequence, based on the ground truth. 19

Table S9: Summary statistics of synteny blocks generated by ntSynt, SibeliaZ [25], halSynteny [26] and SyRI [27] using the human reference genome (T2T build) and one SURVIVOR-simulated rearranged genome. 20

Table S10: Summary of incorrect block counts by tool, sequence divergence in the controlled experiment, and SV type 21

Table S11: Analysis of the genomic regions that are not covered by ntSynt synteny blocks when comparing the human reference genome (T2T build) to one SURVIVOR-rearranged genome sequence assembly with different variant (SNV+indel) rates. 24

Table S12: Expected synteny block statistics for the multi-genome comparisons between the human reference genome (T2T build) and four SURVIVOR-simulated rearranged genomes, based on the ground truth. 24

Table S13: Summary statistics of synteny blocks generated by ntSynt and SibeliaZ using the human reference genome (T2T build) and four SURVIVOR-simulated rearranged genomes. 25

Table S14: Contiguity, coverage and benchmarking statistics for synteny blocks computed on human, bonobo, chimpanzee and gorilla reference genome builds using ntSynt, SibeliaZ, and the SyntenyPortal web application. 25

Table S15: Contiguity, coverage and benchmarking statistics for synteny blocks computed between human, bonobo, chimpanzee and gorilla genome assemblies using ntSynt and SibeliaZ. 26

Table S16: Contiguity, coverage and benchmarking statistics for synteny blocks between human, mouse and rat reference genome assemblies using ntSynt and the SyntenyPortal web application. 26

Table S17: Breakdown of benchmarking statistics for computing synteny blocks between human, mouse and rat reference genomes using ntSynt. 26

Table S18: Contiguity, coverage and benchmarking results from computing synteny blocks between 11 bee genomes of the genus *Andrena* using ntSynt. 27

Supplementary Discussion 28

Supplementary References 28

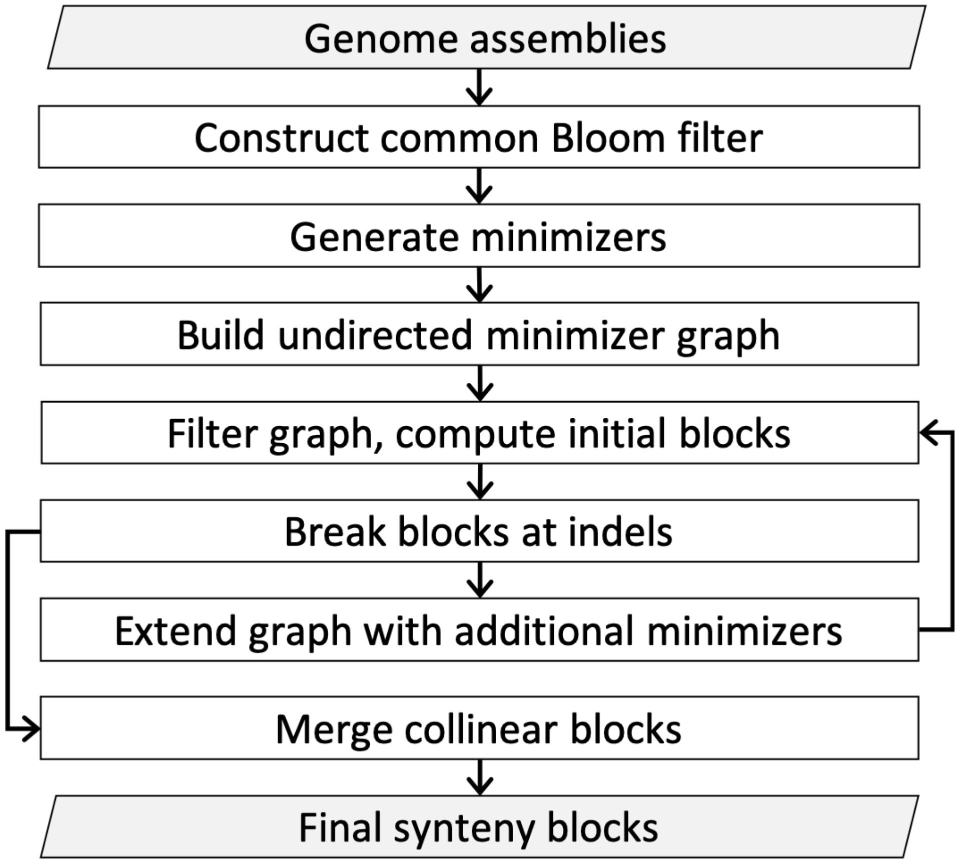

Fig. S1: ntSynt pipeline overview. The initial input for ntSynt is two or more genome assemblies. First, a Bloom filter is built which contains the *k*-mers found in all input genomes. Then, this Bloom filter is used with *indexlr*, a utility found in btllib [1], to generate ordered minimizer sketches for each input genome sequence. An undirected minimizer graph is generated from the minimizer sketches. After this graph is filtered, the initial synteny blocks are computed, followed by breaking the blocks at detected indels. The existing minimizer graph is then extended by generating additional minimizers from regions not covered by the synteny blocks using a lower minimizer window size. Next, synteny blocks are identified from this extended graph using the same filtering methods employed to generate the initial synteny blocks. This graph extension can happen for any number of decreasing window sizes. Following the graph extension rounds, collinear synteny blocks are merged to yield the final synteny blocks.

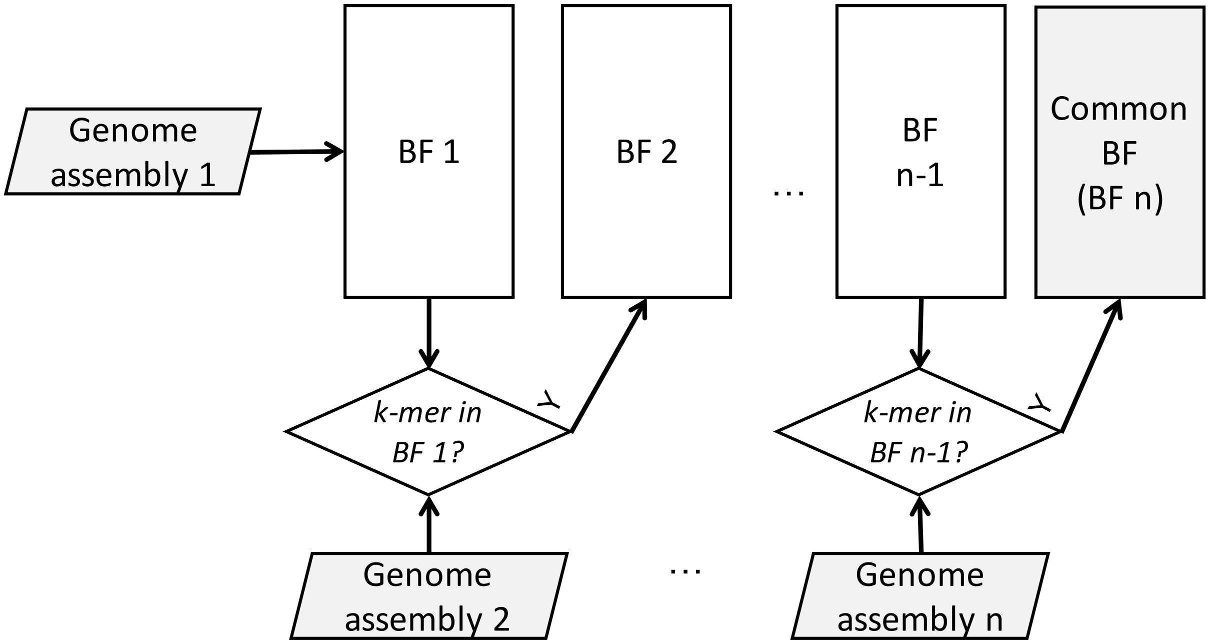

Fig. S2: ntSynt common Bloom filter construction using a cascading approach. To construct the common Bloom filter (BF), which contains the *k*-mers found in each input genome assembly, the *k*-mers from the (arbitrarily chosen) initial genome assembly are first loaded into the level 1 BF (BF1). Then, the next Bloom filter (level 2, BF2) is initialized. The next input genome assembly is *k*-merized using ntHash2 [2], and each *k*-mer queried against the level 1 Bloom filter. All *k*-mers that are present in the level 1 BF are added to the level 2 BF. This process continues for all input sequences, up to input genome assembly *n*, where the *k*-mers from input genome assembly *n* will be queried against BF level *n*-1, and inserted into BF level *n* if present. The level *n* BF is the final common BF, which is then used in subsequent steps. Due to the nature of the cascading approach, only two Bloom filters are kept in memory at a time.

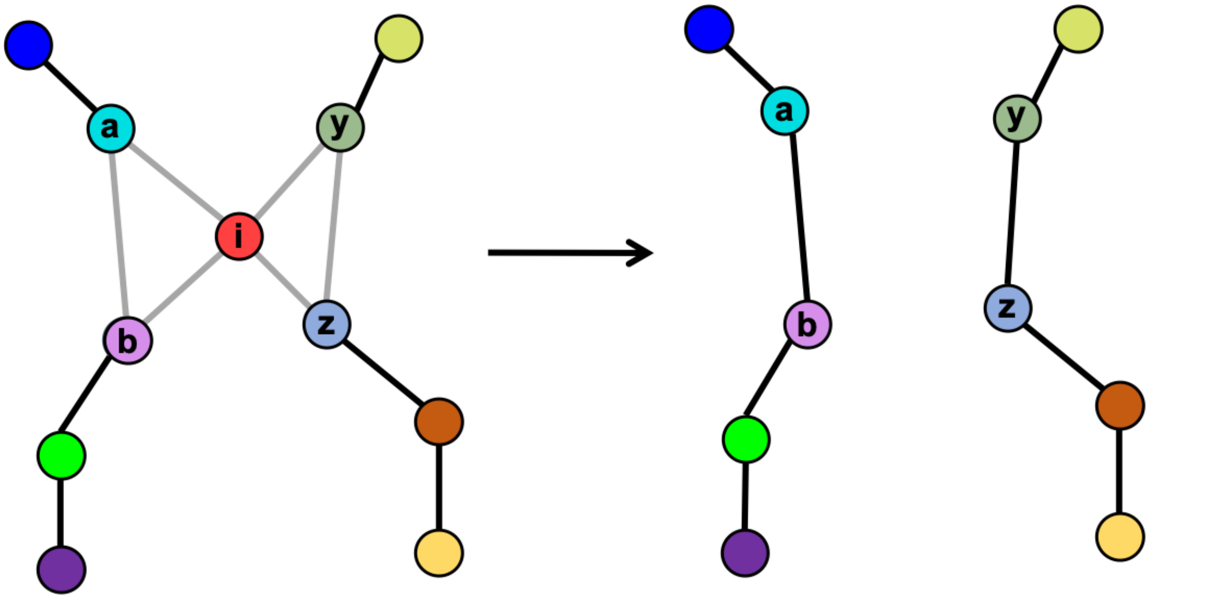

Fig. S3: Simplifying the ntSynt minimizer graph. An example of the graph topology targeted by the graph simplification algorithm is shown. The nodes are minimizers, with different minimizers indicated using varying colours. The edges are coloured based on whether they have full input genome sequence input support (black; edge weight = *n*, where *n* is the number of input genomes) or if there is disagreement between genome sequence inputs (grey; edge weight < *n*). For each partially anchored edge *u, v* (degree(*u*) == degree(*v*) == 3; only one edge incident to both *u* and *v* has edge weight == *n*), the graph is traversed to find any alternate paths between *u* and *v* of length 2. For example, between the start and end nodes of partially anchored edges (*a*, *b*) and (*y*, *z*), there are alternate paths (*a, i, b*) and (*y, i, z*). For these alternate paths, the middle minimizer (*i*) is removed from the graph, and the weights of the direct edges are allocated full input sequence support.

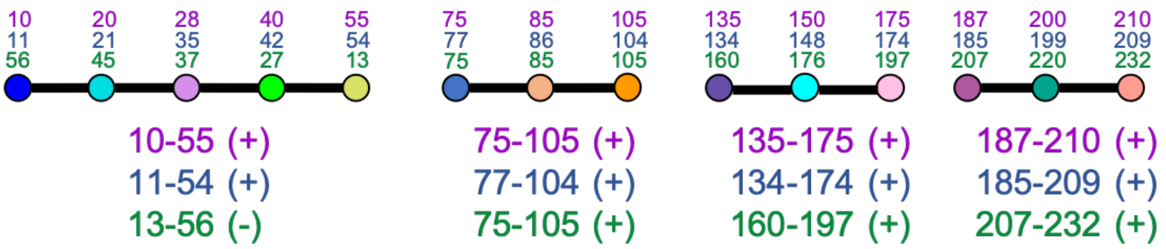

Fig. S4: Converting linear graph paths to synteny block coordinates. Three input genome sequences are being compared here, indicated with purple, blue and green text. Minimizers are shown by the coloured circles, and the numbers above the minimizers indicate the position of the minimizer in the respective input sequence. The ranges and orientations below each block indicate the computed synteny block coordinates and orientations for each genome input in each synteny block.

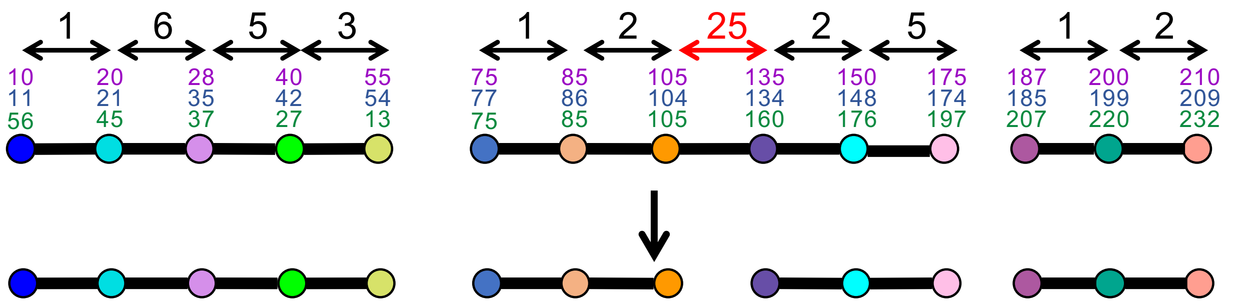

Fig. S5: Indel detection in ntSynt. The positions of the given minimizer in each assembly are indicated by the coloured numbers above the minimizer graph nodes (3 different input sequences indicated by purple, blue and green). For each minimizer graph edge, the interarrival distances for each genome sequence are computed. Then, the indel score is determined from the maximum difference between these interarrival distances. If the indel score is greater than the indel score threshold (--*indel*), that edge is removed from the graph to break the synteny block at the putative indel. In the above example, the indel scores are the numbers above the double-ended arrows. For this example, assuming that the indel score threshold is set at 10, the edge indicated with the red arrow will be removed from the graph, thus breaking the synteny block at this indel.

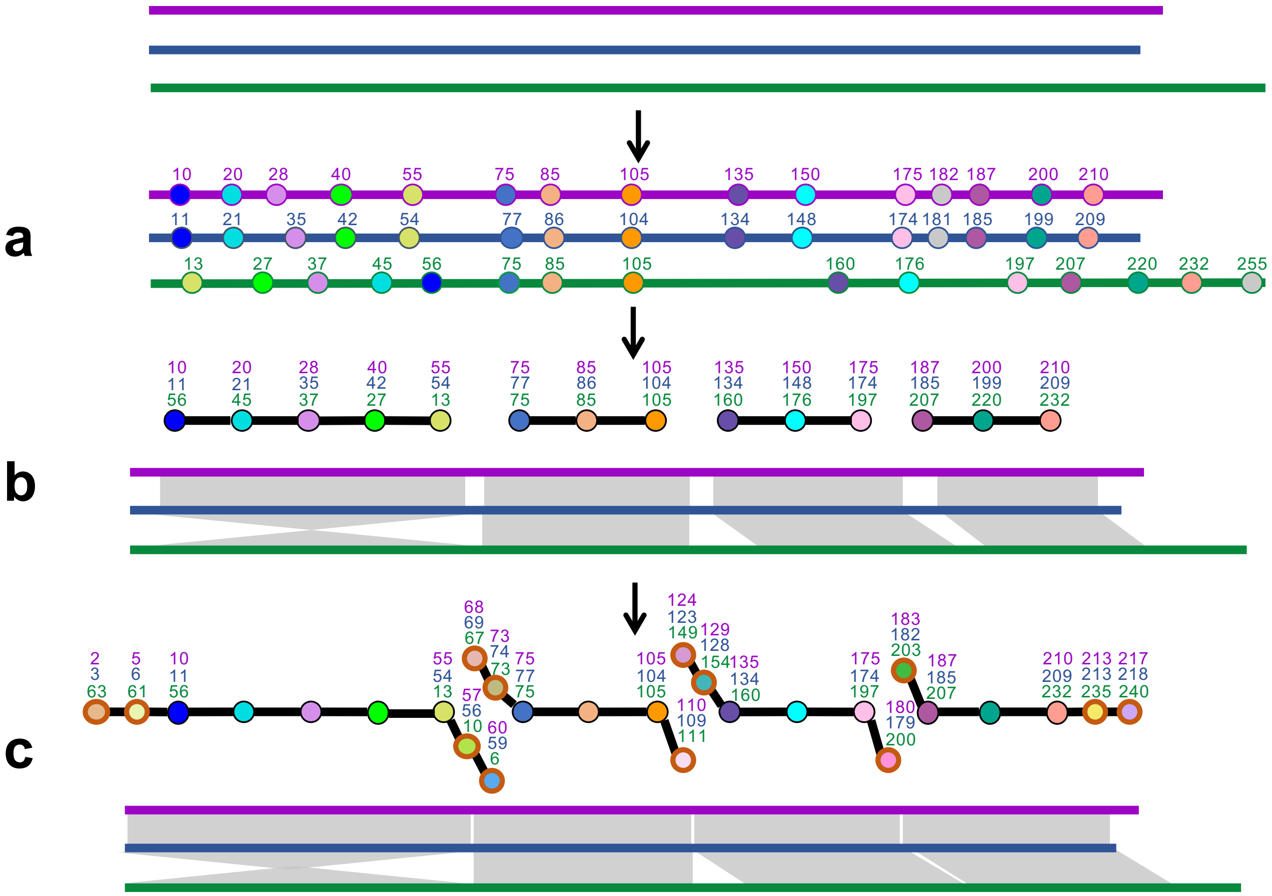

Fig. S6: Extension of ntSynt synteny blocks using minimizers computed with lower window sizes. The positions of the given minimizer in each input genome sequence are indicated by the coloured numbers above the minimizer graph nodes (3 different input genome sequences indicated by purple, blue and green lines). After computing the initial synteny blocks (a-b), including indel detection, the synteny block coordinates can be refined by extending the existing minimizer graph using additional minimizers computed with a lower window size (c). Minimizers from the genomic regions that are not already covered by a synteny block are computed using a lower window size (--*w_rounds*). These minimizers are used to extend the existing graph paths, or synteny blocks, to refine their coordinates. The additional minimizers in (c) are indicated with a thick orange border.

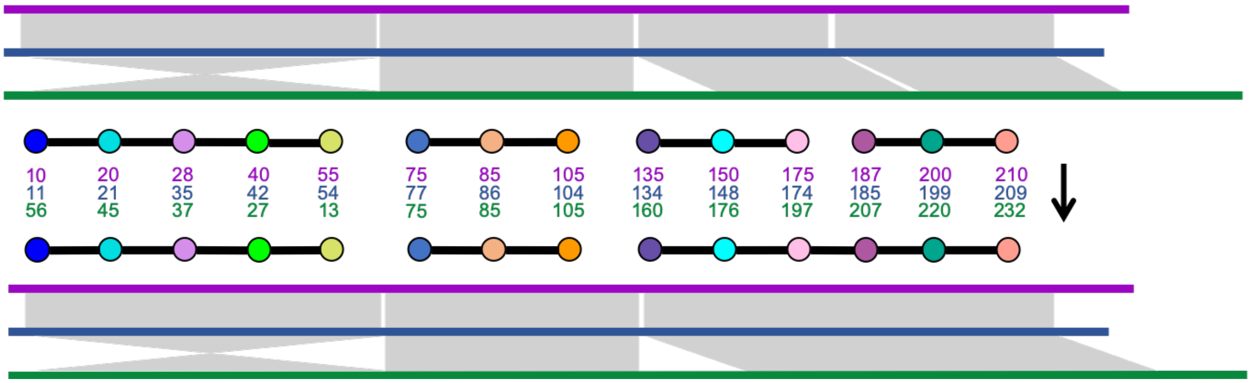

Fig. S7: Merging collinear synteny blocks. Adjacent synteny blocks are considered collinear if they are not separated by an indel, are less than the merge threshold apart, and have consistent contig IDs, orientation and positions.

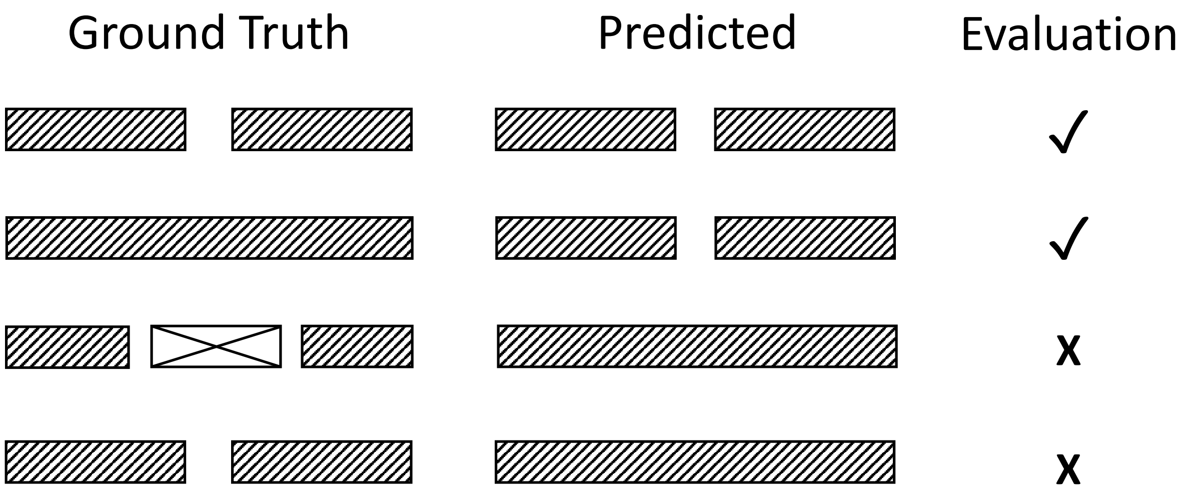

Fig. S8: **Evaluating the correctness (or accuracy) of syntenic sequence blocks on controlled (synthetic) data.** The Predicted genome synteny blocks (right cross-hatched rectangles) are compared to Ground Truth blocks (left cross-hatched rectangles) for four scenarios. A cross-marked rectangle depicts a synteny block in reverse orientation. Evaluation for each scenario is shown on the right-hand side, with check marks and “x” symbols indicating correct and incorrect assessments, respectively.

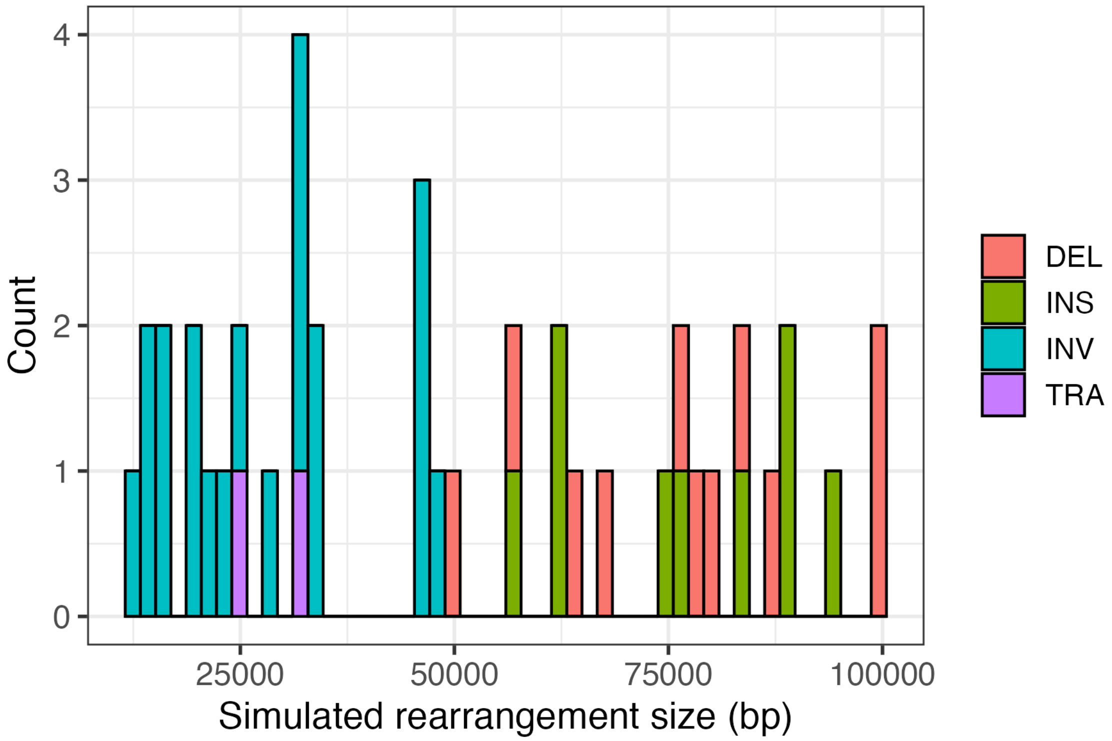

Fig. S9: **Simulated rearrangement size distribution.** Pairwise synteny blocks of various lengths (shown in a stacked bar graph) were derived from comparing a human reference genome (GRCh38) and a simulated rearranged human genome with controlled large indels (DEL: deletion, INS: insertions), inversions (INV), and translocations (TRA).

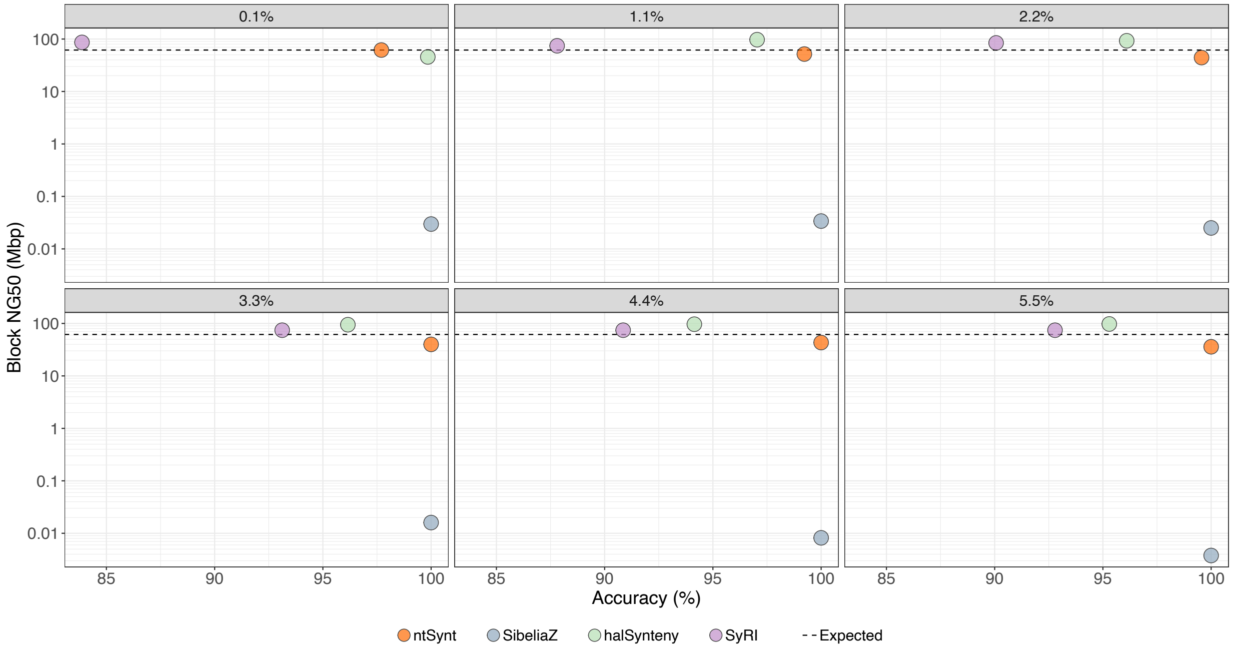

Fig. S10: Synteny block contiguity versus accuracy across increasing divergence levels in simulated human genomes. Rearranged genome sequences were simulated using SURVIVOR [3], with SNVs and indels introduced using pIRS [4] at varying nucleotide sequence divergence rates (0.1-5.5%, facet plots). In each facet plot, synteny block NG50 lengths (y-axis) are plotted against accuracy (x-axis), defined as the percentage of correctly placed blocks ((Total Number of Blocks – Incorrect Blocks based on Fig. S3) ÷ Total Number of Blocks × 100). Each point represents one replicate; jitter was applied for visibility.

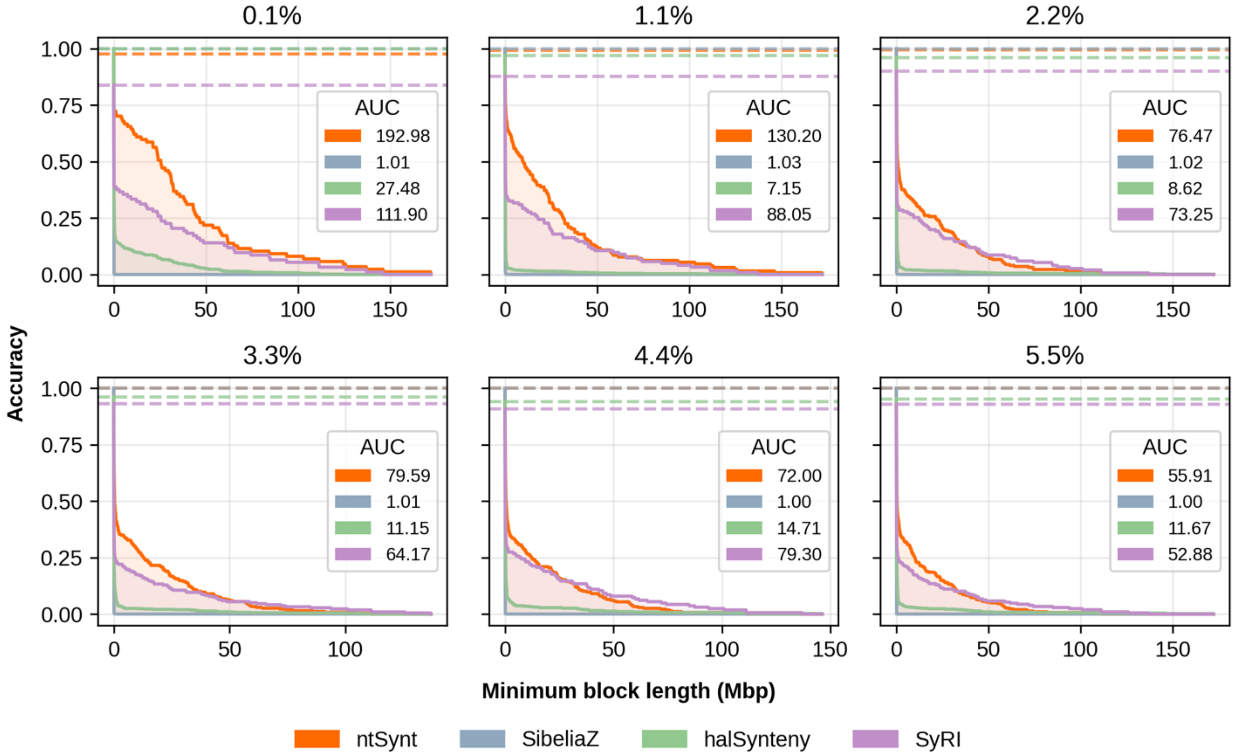

Fig. S11: **Synteny accuracy across different minimum block length thresholds at various simulated sequence divergence rates (0.1-5.5%, facet plots).** Accuracy (y-axis) is defined as the number of correctly identified blocks longer than x Mbp divided by the total number of synteny blocks generated by each tool. Dashed lines indicate the overall accuracy without a length threshold (x = 0). Tools generating highly fragmented blocks result in sharp declines in accuracy at higher thresholds, leading to a reduced area under the curve (AUC).

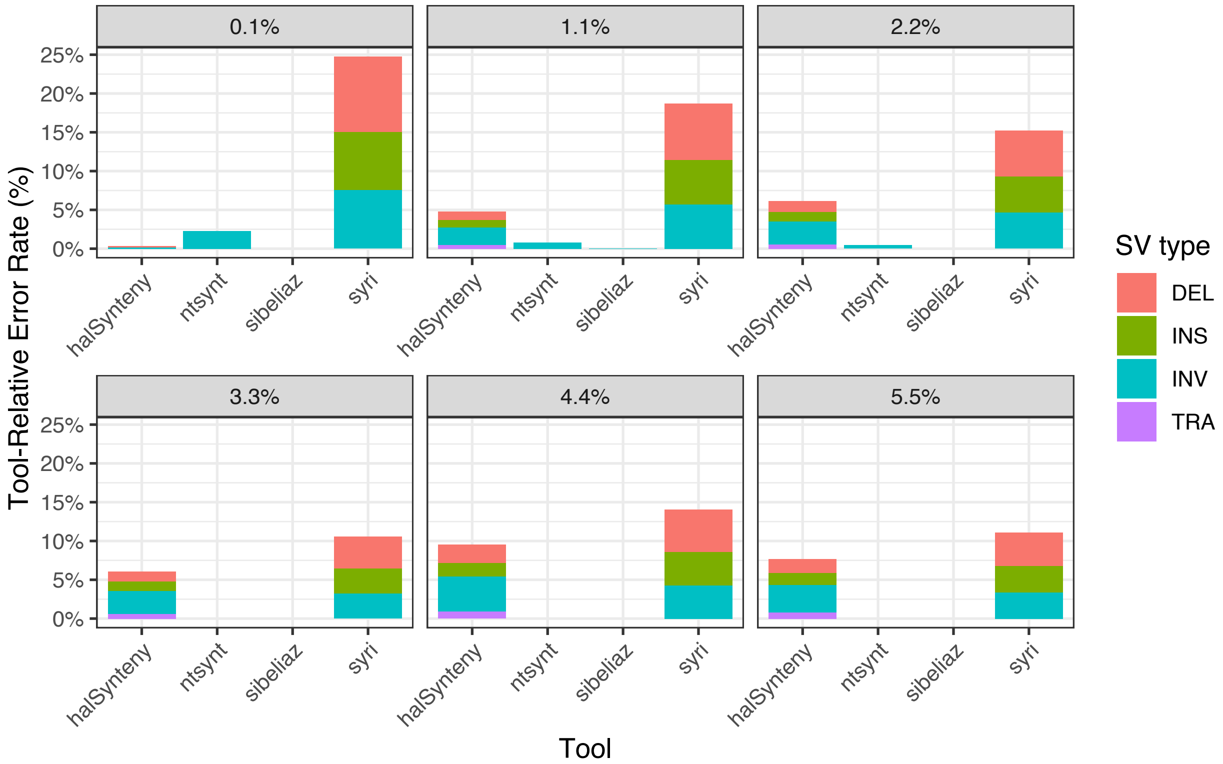

Fig. S12: Tool-relative error rates by simulated structural variant (SV) type across sequence divergence levels (0.1-5.5%, facet plots) in the controlled experiment. Bars indicate the percentage of incorrectly inferred blocks relative to the total number of blocks per tool, stratified by SV type (DEL: deletion, INS: insertion, INV: inversion, TRA: translocation). Blocks assigned to multiple SV types are counted once per type, and therefore may be represented more than once.

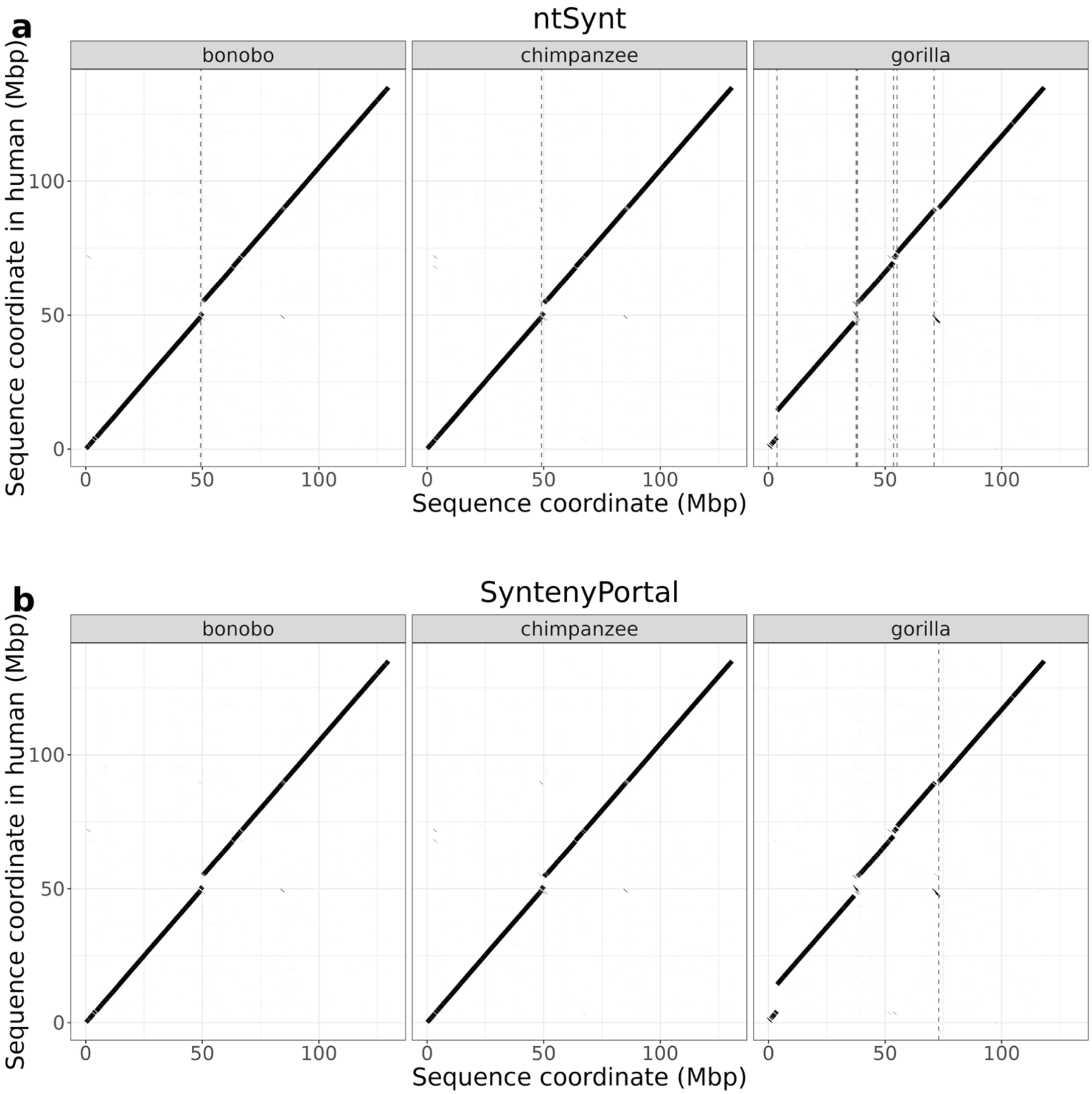

Fig. S13: Pairwise dot plots between human chromosome 11 and the chromosome 11 sequence of three other primate genome assemblies: bonobo, chimpanzee and gorilla. The pairwise alignment blocks were generated using minimap2 [5]. The vertical grey dashed lines denote indels greater than 300 kbp identified by (a) ntSynt or (b) SyntenyPortal [6] in any of the compared primate genomes.

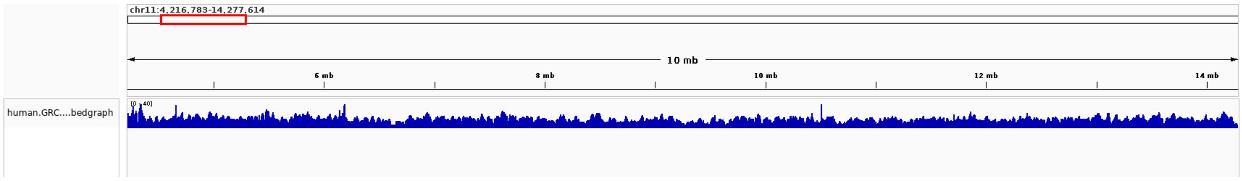

Fig. S14: IGV Screenshot showing alignments of gorilla long reads to the human reference genome. All HiFi sequencing reads were aligned to the human genome reference sequence (GRCh38) using minimap2. The alignments were converted to bedgraph format using bedtools [7] and visualized using IGV [8], zooming into the coordinates corresponding to the putative deletion in the gorilla genome (Kamilah_GGO_v0 chromosome 11, coordinates 4,249,395-14,277,464). The PacBio HiFi reads are available from SRA under accession SRR13446351.

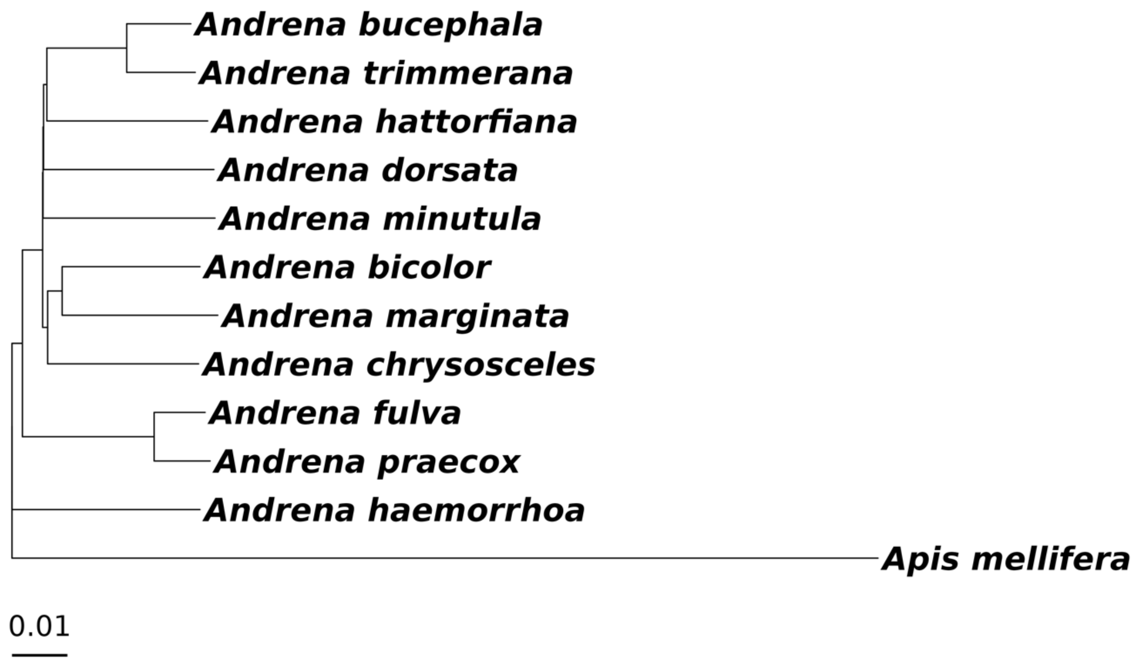

Fig. S15: *Andrena* phylogenetic tree. The phylogenetic tree was generated using Mashtree [9] with the *Andrena* genome assemblies, and rooted using *Apis mellifera* (GCA_000002195.1) as the outgroup. The tree was visualized using ggtree [10].

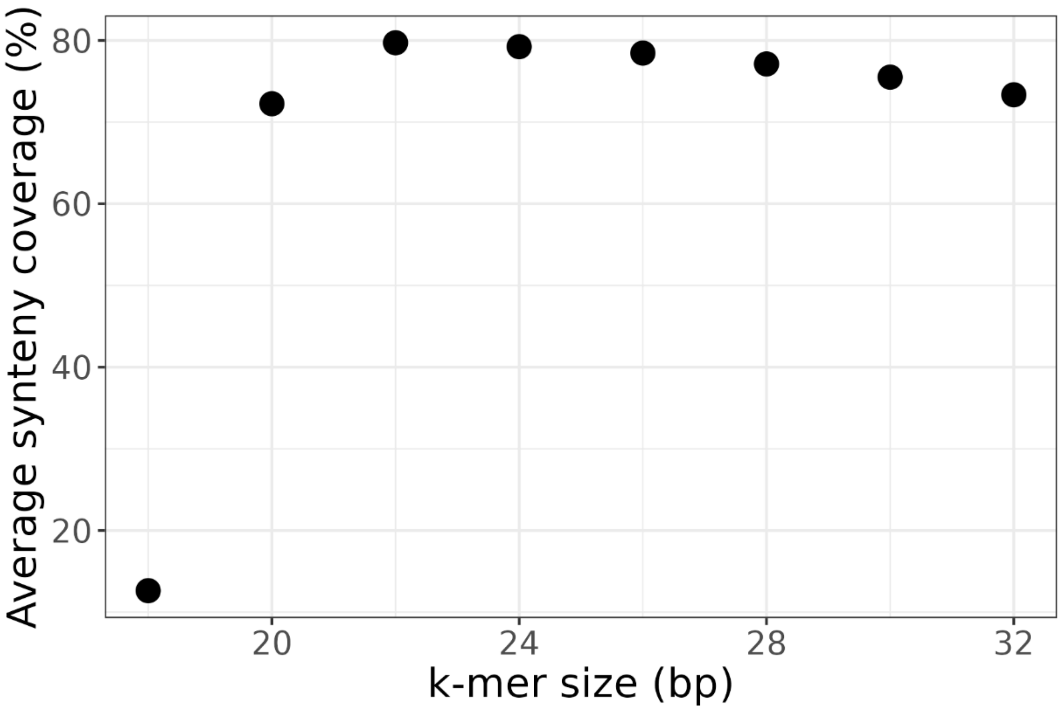

Fig. S16: Average synteny coverage of ntSynt blocks between human, mouse and rat reference genomes, sweeping on the *k*-mer size. Synteny blocks were computed between the reference genomes using the parameters listed in Table S14 with --*indel* 500000, only varying the *k*-mer size for generating the minimizer sketches.

Table S1: Output format of ntSynt synteny blocks file. The output file is in tab-separated format.

Column number	Description
**1**	Synteny block ID - Lines with the same ID are part of the same synteny block
**2**	Genome file name
**3**	Genome chromosome/contig
**4**	Genome start coordinate
**5**	Genome end coordinate
**6**	Strand
**7**	Number of mapped minimizers in this synteny block
**8**	Reason for discontinuity with previous synteny block

Table S2: Default ntSynt parameter settings based on the user-supplied divergence (--*divergence*).

Divergence range	Presets (default parameters)
< 1%	--block_size 500 --indel 10000 --merge 10000 --w_rounds 100 10
1% - 10%	--block_size 1000 --indel 50000 --merge 100000 --w_rounds 250 100
>10%	--block_size 10000 --indel 100000 --merge 1000000 --w_rounds 500 250

Table S3: Parameter settings used when running synteny block comparator tools.

Tool	Sequence divergence	Parameters
SibeliaZ<br>(+ maf2synteny)	< 1%	*SibeliaZ*: -n -t12<br>*maf2synteny*: -b 500
	>= 1%	*SibeliaZ*: -n -t12<br>*maf2synteny*: -b 1000
halSynteny<br>(+ Progressive Cactus)	< 1%	*Progressive Cactus*: --maxCores 12 --binariesMode local<br>*halSynteny*: --minBlockSize 500 --maxAnchorDistance 10000
	>= 1%	*Progressive Cactus*: --maxCores 12 --binariesMode local<br>*halSynteny*: --minBlockSize 1000 --maxAnchorDistance 100000
SyRI<br>(+ minimap2)	< 1%	*minimap2*: -x asm -t 12 -c --eqx<br>*SyRI*: --nosnp --nc 12 -F P --invgaplen 10000 --tdgaplen 10000<br>--no-chrmatch
	>= 1%	*minimap2*: -x asm -t 12 -c --eqx<br>*SyRI*: --nosnp --nc 12 -F P --invgaplen 100000 --tdgaplen 100000<br>--no-chrmatch

Table S4: Reference genome assemblies used for synteny block analysis with ntSynt, SibeliaZ and SyntenyPortal.

Species	Common name	Build	Accession
Homo sapiens	Human	GRCh38	GCA_000001405.15
Pan paniscus	Bonobo	Mhudiblu_PPA_v0	GCF_013052645.1
Pan troglodytes	Chimpanzee	Clint_PTRv2	GCF_002880755.1
Gorilla gorilla gorilla	Gorilla	Kamilah_GGO_v0	GCF_008122165.1
Mus musculus	Mouse	GRCm39	GCA_000001635.9
Rattus norvegicus	Rat	Rnor_6.0	GCA_000001895.4

Table S5: Mapped read statistics from aligning gorilla PacBio HiFi reads (individual Kamilah) to the gorilla reference genome build used in the synteny tests (Kamilah_GGO_v0) and a newer gorilla reference genome build (mGorGor1). All HiFi sequencing reads were aligned to the human genome reference sequence (GRCh38) using minimap2, and the reads that aligned to a putative deletion in the gorilla genome (Kamilah_GGO_v0 chromosome 11, coordinates 4,249,395-14,277,464) were extracted using samtools [11]. The extracted reads were then aligned to the two gorilla genome builds using minimap2, and filtered to retain primary mapped reads with mapping quality >= 50. The PacBio HiFi reads are available from SRA under accession SRR13446351 and the mGorGor1 assembly is available from GenBank under accession GCA_029281585.1.

Assembly build	Total number of reads	Number of mapped reads
**Kamilah_GGO_v0**	7,212	185 (2.6%)
**mGorGor1**	7,212	7,207 (99.9%)

Table S6: Genome sequence assemblies used for synteny block analysis with ntSynt and SibeliaZ.

Species	Common name	Cell line /individual	Assembler	Accession
Homo sapiens	Human	NA24385	GoldRush [12]	https://doi.org/10.5281/zenodo.7884681
Pan paniscus	Bonobo	Mhudiblu	hifiasm [13]	GCA_030221875.1
Pan troglodytes	Chimpanzee	Clint	hifiasm	GCA_030128855.1
Gorilla gorilla gorilla	Gorilla	Kamilah	hifiasm	GCA_030174185.1

Table S7: Genome assemblies from the genus *Andrena* analyzed using ntSynt. All genome sequences are assembled at chromosome-level and were generated as part of the Darwin Tree of Life project under the Earth BioGenome umbrella (PRJNA533106).

Assembly Accession	Reference	Species	Assembly size (Mbp)	Number of chromosomes
GCA_929108735.1	[14]	*Andrena dorsata*	269.1	3
GCA_963855975.1	[15]	*Andrena chrysosceles*	356.6	4
GCA_947577245.1	[16]	*Andrena bucephala*	379.5	5
GCA_951215215.1	[17]	*Andrena trimmerana*	344.0	5
GCA_960531205.1	[18]	*Andrena bicolor*	247.2	5
GCA_963932335.1	[19]	*Andrena marginata*	297.2	6
GCA_946251845.1	[20]	*Andrena fulva*	370.5	7
GCA_963932275.1	[21]	*Andrena praecox*	442.8	7
GCA_944738655.2	[22]	*Andrena hattorfiana*	421.4	7
GCA_929113495.1	[23]	*Andrena minutula*	349.9	7
GCA_910592295.1	[24]	*Andrena haemorrhoa*	291.3	7

Table S8: Expected synteny block statistics for the pairwise comparisons between the human reference genome (T2T build) and one SURVIVOR [3]-simulated rearranged genome sequence, based on the ground truth. The expected values of these statistics are the same for each rate of SNV and indels introduced with pIRS [4].

Number of blocks	Synteny coverage (%)	Block NG50 length (Mbp)
91	100.00	61.71

Table S9: Summary statistics of synteny blocks generated by ntSynt, SibeliaZ [25], halSynteny [26] and SyRI [27] using the human reference genome (T2T build) and one SURVIVOR-simulated rearranged genome. SNVs and indels were introduced into the same SURVIVOR rearranged genome sequence at various SNV and indel rates, using pIRS. The synteny coverage and block NG50 length statistics are averaged for the two input genome sequence assemblies. Accuracy was calculated as the percentage of correctly placed synteny blocks, defined as (Total Number of Blocks – Incorrect Blocks based on Fig. S3) ÷ Total Number of Blocks × 100. The wall-clock time and memory usage statistics shown include any mapping steps required prior to running the tool (minimap2 [5] for SyRI and Progressive Cactus [28] for halSynteny). The benchmarks were averaged over triplicate runs, with the average ± standard deviation tallied in the table.

SNV rate (%)	Indel rate (%)	Tool	Number of blocks	Synteny cov-erage (%)	Block NG50 length (Mbp)	Acc-uracy (%)	Wall-clock time (min)	Peak memory (GB)
0.1	0.01	**ntSynt**	87	100.00	61.71	97.70	26.78 ± 4.33	33.99 ± 0.1
		**SibeliaZ**	231,556	89.84	0.03	100.00	76.61 ± 7.71	31.50 ± 0.0
		**halSynteny**	655	98.94	45.61	99.85	8,519.27 ± 361.45	17.56 ± 0.0
		**SyRI**	93	100.02	86.30	83.87	19.00 ± 0.52	24.60 ± 0.0
1.0	0.10	**ntSynt**	129	99.29	51.73	99.22	26.09 ± 2.83	33.94 ± 0.1
		**SibeliaZ**	142,318	86.93	0.03	100.00	113.34 ± 11.79	45.90 ± 0.0
		**halSynteny**	879	99.73	97.22	97.04	8,535.16 ± 357.66	22.88 ± 6.9
		**SyRI**	123	100.00	74.29	87.80	25.00 ± 1.05	23.92 ± 0.1
2.0	0.20	**ntSynt**	226	96.90	44.30	99.56	37.37 ± 2.39	33.90 ± 0.1
		**SibeliaZ**	172,172	85.74	0.03	100.00	147.06 ± 14.82	54.69 ± 0.0
		**halSynteny**	718	99.77	92.60	96.10	8,517.66 ± 345.14	29.74 ± 2.1
		**SyRI**	151	99.97	84.34	90.07	32.14 ± 1.07	22.30 ± 0.0
3.0	0.30	**ntSynt**	269	95.44	39.89	100.00	49.94 ± 8.77	33.91 ± 0.1
		**SibeliaZ**	234,919	83.88	0.02	100.00	195.36 ± 20.32	58.68 ± 0.0
		**halSynteny**	676	99.58	94.57	96.15	8,524.84 ± 346.54	35.04 ± 0.1
		**SyRI**	218	99.92	74.29	93.12	37.80 ± 1.52	21.70 ± 0.0
4.0	0.40	**ntSynt**	277	94.80	43.21	100.00	61.49 ± 13.14	33.83 ± 0.2
		**SibeliaZ**	358,553	80.73	0.01	100.00	267.79 ± 12.64	59.62 ± 0.0
		**halSynteny**	461	98.80	96.69	96.14	8,511.31 ± 357.38	44.02 ± 0.8
		**SyRI**	164	99.97	74.29	90.85	61.31 ± 2.55	29.35 ± 0.1
5.0	0.50	**ntSynt**	302	94.25	35.91	100.00	72.17 ± 13.82	33.91 ± 0.2
		**SibeliaZ**	545,068	74.39	0.00	100.00	339.58 ± 33.23	58.68 ± 0.0
		**halSynteny**	510	99.63	97.68	95.29	8,528.08 ± 359.78	55.81 ± 4.5
		**SyRI**	208	99.95	74.29	92.79	64.64 ± 2.24	27.54 ± 0.1

Table S10: Summary of incorrect block counts by tool, sequence divergence in the controlled experiment, and SV type. For each combination, the table reports the number of incorrectly inferred blocks, the total number of blocks, and the tool-relative error rate (%). Blocks assigned to multiple SV (structural variant) types are counted once per type, and therefore may be represented more than once. DEL: deletion; INS: insertion; INV: inversion; TRA: translocation.

Tool	Divergence (%)	Total blocks	SV type	Number of incorrect blocks	Tool-Relative Error Rate (%)
**ntSynt**	0.1	87	DEL	0	0.0000
			INS	0	0.0000
			INV	2	2.2989
			TRA	0	0.0000
**SibeliaZ**		231,556	DEL	0	0.0000
			INS	0	0.0000
			INV	0	0.0000
			TRA	0	0.0000
**halSynteny**		655	DEL	1	0.1527
			INS	0	0.0000
			INV	1	0.1527
			TRA	0	0.0000
**SyRI**		93	DEL	9	9.6774
			INS	7	7.5269
			INV	7	7.5269
			TRA	0	0.0000
**ntSynt**	1.1	129	DEL	0	0.0000
			INS	0	0.0000
			INV	1	0.7752
			TRA	0	0.0000
**SibeliaZ**		142,318	DEL	0	0.0000
			INS	1	0.0007
			INV	1	0.0007
			TRA	0	0.0000
**halSynteny**		879	DEL	10	1.1377
			INS	8	0.9101
			INV	20	2.2753
			TRA	4	0.4551
**SyRI**		123	DEL	9	7.3171
			INS	7	5.6911
			INV	7	5.6911

			TRA	0	0.0000
**ntSynt**	2.2	226	DEL	0	0.0000
			INS	0	0.0000
			INV	1	0.4425
			TRA	0	0.0000
**SibeliaZ**		172,172	DEL	0	0.0000
			INS	0	0.0000
			INV	0	0.0000
			TRA	0	0.0000
**halSynteny**		718	DEL	10	1.3928
			INS	9	1.2535
			INV	21	2.9248
			TRA	4	0.5571
**SyRI**		151	DEL	9	5.9603
			INS	7	4.6358
			INV	7	4.6358
			TRA	0	0.0000
**ntSynt**	3.3	269	DEL	0	0.0000
			INS	0	0.0000
			INV	0	0.0000
			TRA	0	0.0000
**SibeliaZ**		234,919	DEL	0	0.0000
			INS	1	0.0004
			INV	1	0.0004
			TRA	0	0.0000
**halSynteny**		676	DEL	9	1.3314
			INS	8	1.1834
			INV	20	2.9586
			TRA	4	0.5917
**SyRI**		218	DEL	9	4.1284
			INS	7	3.2110
			INV	7	3.2110
			TRA	0	0.0000
**ntSynt**	4.4	277	DEL	0	0.0000
			INS	0	0.0000
			INV	0	0.0000
			TRA	0	0.0000
**SibeliaZ**		358,553	DEL	0	0.0000
			INS	0	0.0000

			INV	0	0.0000
			TRA	0	0.0000
**halSynteny**		461	DEL	11	2.3861
			INS	8	1.7354
			INV	21	4.5553
			TRA	4	0.8677
**SyRI**		164	DEL	9	5.4878
			INS	7	4.2683
			INV	7	4.2683
			TRA	0	0.0000
**ntSynt**	5.5	302	DEL	0	0.0000
			INS	0	0.0000
			INV	0	0.0000
			TRA	0	0.0000
**SibeliaZ**		545,068	DEL	0	0.0000
			INS	1	0.0002
			INV	0	0.0000
			TRA	0	0.0000
**halSynteny**		510	DEL	9	1.7647
			INS	8	1.5686
			INV	18	3.5294
			TRA	4	0.7843
**SyRI**		208	DEL	9	4.3269
			INS	7	3.3654
			INV	7	3.3654
			TRA	0	0.0000

Table S11: Analysis of the genomic regions that are not covered by ntSynt synteny blocks when comparing the human reference genome (T2T build) to one SURVIVOR-rearranged genome sequence assembly with different variant (SNV+indel) rates. The regions of the reference genome sequence that were not covered by synteny blocks were compared with annotated centromere coordinates.

SNV rate (%)	Indel rate (%)	Centromeric?	Percentage of genome without synteny block coverage (%)
0.1	0.01	No	0.03
		Yes	0.00
1.0	0.10	No	0.03
		Yes	0.71
2.0	0.20	No	0.04
		Yes	3.08
3.0	0.30	No	0.16
		Yes	4.43
4.0	0.40	No	0.19
		Yes	5.03
5.0	0.50	No	0.27
		Yes	5.50

Table S12: Expected synteny block statistics for the multi-genome comparisons between the human reference genome (T2T build) and four SURVIVOR-simulated rearranged genomes, based on the ground truth. The expected values of the statistics are the same for each rate of SNV and indels introduced using pIRS.

Number of blocks	Synteny coverage (%)	Block NG50 length (Mbp)
295	100.00	24.59

Table S13: Summary statistics of synteny blocks generated by ntSynt and SibeliaZ using the human reference genome (T2T build) and four SURVIVOR-simulated rearranged genomes. SNVs and indels were introduced into the SURVIVOR rearranged genomes at various rates using pIRS. The synteny coverage and block NG50 length statistics are averaged for the five input genome sequence assemblies. For the block NG50 length statistics, "-" denotes where this statistic could not be calculated due the synteny coverage being less than half of the input genome size. The benchmarks were averaged over triplicate runs, with the average ± standard deviation tallied in the table.

Tool	SNV rate (%)	Indel rate (%)	Number of blocks	Synteny coverage (%)	Block NG50 length (Mbp)	Wall-clock time (h)	Peak memory (GB)
**ntSynt**	0.1	0.01	290	99.87	24.59	1.03 ± 0.29	34.05 ± 0.02
**ntSynt**	1.0	0.10	315	98.67	23.73	1.25 ± 0.54	34.01 ± 0.06
**ntSynt**	2.0	0.20	384	95.26	21.97	1.23 ± 0.53	33.95 ± 0.05
**ntSynt**	3.0	0.30	466	93.24	20.34	1.33 ± 0.60	34.01 ± 0.05
**ntSynt**	4.0	0.40	485	92.12	18.36	1.29 ± 0.63	33.98 ± 0.02
**ntSynt**	5.0	0.50	477	91.11	16.78	1.28 ± 0.54	34.02 ± 0.04
**SibeliaZ**	0.1	0.01	163,945	90.78	0.05	2.01 ± 0.29	64.46 ± 0.02
**SibeliaZ**	1.0	0.10	169,377	85.98	0.03	4.67 ± 1.06	142.06 ± 0.06
**SibeliaZ**	2.0	0.20	392,391	76.28	0.01	9.44 ± 1.80	179.87 ± 0.04
**SibeliaZ**	3.0	0.30	557,286	45.94	-	20.45 ± 4.19	194.17 ± 0.01
**SibeliaZ**	4.0	0.40	159,549	9.50	-	39.06 ± 7.18	195.58 ± 0.00
**SibeliaZ**	5.0	0.50	20,947	0.76	-	58.17 ± 13.11	189.70 ± 0.02

Table S14: Contiguity, coverage and benchmarking statistics for synteny blocks computed on human, bonobo, chimpanzee and gorilla reference genome builds using ntSynt, SibeliaZ, and the SyntenyPortal web application. Synteny coverage of complete blocks refers to synteny blocks that include all four input genome assemblies. SyntenyPortal is a web application based on pre-computed sequence alignments, therefore benchmarks cannot be computed for this resource.

Tool	Number of synteny blocks	Synteny coverage (%)	Synteny coverage of complete blocks (%)	NG50 block length (Mbp)	Wall-clock time (h)	Peak memory (GB)
**ntSynt**	1,951	92.23	92.23	7.61	0.80	32.19
**SibeliaZ**	136,961	88.63	85.35	0.05	2.71	87.67
**SyntenyPortal**	186	94.69	94.69	48.14	N/A	N/A

Table S15: Contiguity, coverage and benchmarking statistics for synteny blocks computed between human, bonobo, chimpanzee and gorilla genome assemblies using ntSynt and SibeliaZ. Synteny coverage of complete blocks refers to synteny blocks that include all four input genome sequence assemblies. The human genome assembly was generated using GoldRush, while the other assemblies were produced using hifiasm.

Tool	Number of synteny blocks	Synteny coverage (%)	Synteny coverage of complete blocks (%)	NG50 block length (Mbp)	Wall-clock time (h)	Peak memory (GB)
**ntSynt**	7,450	82.81	82.81	1.30	1.18	32.76
**SibeliaZ**	145,678	83.29	79.13	0.05	3.24	102.64

Table S16: Contiguity, coverage and benchmarking statistics for synteny blocks between human, mouse and rat reference genome assemblies using ntSynt and the SyntenyPortal web application. Synteny coverage of complete blocks refers to synteny blocks that include all three input genome assemblies. SyntenyPortal is a web application based on pre-computed sequence alignments, therefore benchmarks cannot be computed for this resource.

Tool	Number of synteny blocks	Synteny coverage (%)	Synteny coverage of complete blocks (%)	NG50 block length (Mbp)	Wall-clock time (h)	Peak memory (GB)
**ntSynt**	1,069	79.24	79.24	3.86	1.08	34.22
**SyntenyPortal**	806	89.79	89.79	7.05	N/A	N/A

Table S17: Breakdown of benchmarking statistics for computing synteny blocks between human, mouse and rat reference genomes using ntSynt. The ntSynt stages were grouped into three main steps. Note that some processes within the minimizer computation step can run concurrently, thus the sum of times over all steps will be greater than the total wall-clock time of the entire process.

ntSynt step	Wall-clock time (min)	Peak memory (GB)
**common BF construction**	5.38	34.22
**minimizer computation**	18.98	18.13
**synteny block construction**	46.95	25.31

Table S18: Contiguity, coverage and benchmarking results from computing synteny blocks between 11 bee genomes of the genus *Andrena* using ntSynt. The average synteny coverage averages the synteny coverage over all genomes with varying sizes.

Number of synteny blocks	Average synteny coverage (%)	Synteny coverage of smallest genome (%)	N50 block length (kbp)	Wall-clock time (min)	Peak memory (GB)
1,323	69.9	85.3	481.1	14.7	4.0

Supplementary Discussion

To check if a given *k*-mer is present in the Bloom filter, a hash value for the *k*-mer is determined using ntHash [2], and this memory location is queried for a 1 (presence) or 0 (absence). When using this approach, false positives are possible due to hash collisions. It is this data structure-level false positive rate that we are referring to in our study, and not errors in the synteny block detection itself. The latter is evaluated separately in our benchmarking experiments. The common Bloom filter employed by ntSynt is built using a cascading approach, which ensures that the peak memory usage is capped at twice the Bloom filter size. As this size is calculated based on the genome sizes and specified false positive rate, the memory can be further lowered by increasing the false positive rate of the probabilistic Bloom filter data structure. However, this is a balance, as increasing the false positive rate too aggressively can negatively impact the multi-genome mapping sensitivity.

Supplementary References

1. Nikolić V, Kazemi P, Coombe L, Wong J, Afshinfard A, Chu J, et al. btllib: A C++ library with Python interface for efficient genomic sequence processing. Journal of Open Source Software. 2022;7:4720.

2. Kazemi P, Wong J, Nikolić V, Mohamadi H, Warren RL, Birol I. ntHash2: recursive spaced seed hashing for nucleotide sequences. Bioinformatics. 2022;38:4812–3. https://doi.org/10.1093/bioinformatics/btac564

3. Jeffares DC, Jolly C, Hoti M, Speed D, Shaw L, Rallis C, et al. Transient structural variations have strong effects on quantitative traits and reproductive isolation in fission yeast. Nature Communications. 2017;8:14061. https://doi.org/10.1038/ncomms14061

4. Hu X, Yuan J, Shi Y, Lu J, Liu B, Li Z, et al. pIRS: Profile-based Illumina pair-end reads simulator. Bioinformatics. 2012;28:1533–5. https://doi.org/10.1093/bioinformatics/bts187

5. Li H. Minimap2: pairwise alignment for nucleotide sequences. Bioinformatics. 2018;34:3094–100. https://doi.org/10.1093/bioinformatics/bty191

6. Lee J, Hong W, Cho M, Sim M, Lee D, Ko Y, et al. Synteny Portal: a web-based application portal for synteny block analysis. Nucleic Acids Research. 2016;44:W35–40. https://doi.org/10.1093/nar/gkw310

7. Quinlan AR, Hall IM. BEDTools: a flexible suite of utilities for comparing genomic features. Bioinformatics. 2010;26:841–2. https://doi.org/10.1093/bioinformatics/btq033

8. Robinson JT, Thorvaldsdóttir H, Winckler W, Guttman M, Lander ES, Getz G, et al. Integrative genomics viewer. Nature Biotechnology. 2011;29:24–6. https://doi.org/10.1038/nbt.1754

9. Katz LS, Griswold T, Morrison SS, Caravas JA, Zhang S, den Bakker HC, et al. Mashtree: a rapid comparison of whole genome sequence files. Journal of Open Source Software. 2019;4:1762.

10. Yu G, Smith DK, Zhu H, Guan Y, Lam TT. ggtree: an R package for visualization and annotation of phylogenetic trees with their covariates and other associated data. Methods in Ecology and Evolution. Wiley Online Library; 2017;8:28–36.

11. Danecek P, Bonfield JK, Liddle J, Marshall J, Ohan V, Pollard MO, et al. Twelve years of SAMtools and BCFtools. Gigascience. United States; 2021;10. https://doi.org/10.1093/gigascience/giab008

12. Wong J, Coombe L, Nikolić V, Zhang E, Nip KM, Sidhu P, et al. Linear time complexity de novo long read genome assembly with GoldRush. Nature Communications. 2023;14:2906. https://doi.org/10.1038/s41467-023-38716-x

13. Cheng H, Concepcion GT, Feng X, Zhang H, Li H. Haplotype-resolved de novo assembly using phased assembly graphs with hifiasm. Nature Methods. 2021;18:170–5. https://doi.org/10.1038/s41592-020-01056-5

14. Falk S, Mulley JF, of Oxford U, Lab WWGA, of Life WSIT, Darwin Tree of Life Consortium. The genome sequence of the short-fringed mining bee, Andrena dorsata (Kirby, 1802). Wellcome Open Research. 2023;8:373.

15. Crowley LM, University of Oxford and Wytham Woods Genome Acquisition Lab, Darwin Tree of Life Barcoding collective, Wellcome Sanger Institute Tree of Life Management S and L team, Wellcome Sanger Institute Scientific Operations: Sequencing Operations, Wellcome Sanger Institute Tree of Life Core Informatics team, et al. The genome sequence of the Hawthorn Mining Bee, Andrena chrysosceles (Kirby, 1802). Wellcome Open Research. F1000 Research Limited London, UK; 2025;10:100.

16. Crowley LM, of Oxford U, Lab WWGA, of Life WSIT, Darwin Tree of Life Consortium. The genome sequence of the big-headed mining bee, Andrena bucephala (Stephens, 1846). Wellcome Open Research. 2024;9:111.

17. Baker E, Crowley LM, Falk S, University of Oxford and Wytham Woods Genome Acquisition Lab, Darwin Tree of Life Barcoding collective, Wellcome Sanger Institute Tree of Life Management S and L team, et al. The genome sequence of Trimmer's Mining Bee, Andrena trimmerana (Kirby, 1802). Wellcome Open Research. F1000 Research Limited London, UK; 2024;9:691.

18. Falk S, Monks J, of Oxford U, Lab WWGA, of Life WSIT, Darwin Tree of Life Consortium. The genome sequence of Gwynne's mining bee, Andrena bicolor Fabricius, 1775. Wellcome Open Research. 2024;9:140.

19. Mitchell R, Monks J, of Life WSIT, Darwin Tree of Life Consortium. The genome sequence of the Small Scabious Mining Bee, Andrena marginata Fabricius, 1776. Wellcome Open Research. 2024;9:447.

20. Crowley LM, Mulley JF, of Oxford U, Lab WWGA, of Life WSIT, Darwin Tree of Life Consortium. The genome sequence of the Tawny Mining Bee, Andrena fulva (Müller, 1766). Wellcome Open Research. 2023;8:258.

21. Crowley LM, University of Oxford and Wytham Woods Genome Acquisition Lab, Darwin Tree of Life Barcoding collective, Wellcome Sanger Institute Tree of Life Management S and L team, Wellcome Sanger Institute Scientific Operations: Sequencing Operations, Wellcome Sanger Institute Tree of Life Core Informatics team, et al. The genome sequence of the Small Sallow Mining Bee, Andrena praecox (Scopoli, 1763). Wellcome Open Research. F1000 Research Limited London, UK; 2025;10:290.

22. Falk S, Tan K-T, of Oxford U, Lab WWGA, of Life WSIT, Darwin Tree of Life Consortium. The genome sequence of the Large Scabious Mining Bee, Andrena hattorfiana (Fabricius, 1775). Wellcome Open Research. 2023;8:224.

23. Falk S, University of Oxford and Wytham Woods Genome Acquisition Lab, Darwin Tree of Life Barcoding collective, Wellcome Sanger Institute Tree of Life programme, Wellcome Sanger Institute Scientific Operations: DNA Pipelines collective, Tree of Life Core Informatics collective, et al. The genome sequence of the common mini-mining bee Andrena minutula (Kirby, 1802). Wellcome Open Research. F1000 Research Limited London, UK; 2022;7:300.

24. Crowley LM, of Oxford U, Lab WWGA, of Life WSIT, Darwin Tree of Life Consortium. The genome sequence of the Orange-tailed Mining Bee, Andrena haemorrhoa (Fabricius, 1781). Wellcome Open Research. 2023;8:396.

25. Minkin I, Medvedev P. Scalable multiple whole-genome alignment and locally collinear block construction with SibeliaZ. Nature Communications. 2020;11:6327. https://doi.org/10.1038/s41467-020-19777-8

26. Krasheninnikova K, Diekhans M, Armstrong J, Dievskii A, Paten B, O'Brien S. halSynteny: a fast, easy-to-use conserved synteny block construction method for multiple whole-genome alignments. GigaScience. Oxford University Press; 2020;9:giaa047.

27. Goel M, Sun H, Jiao W-B, Schneeberger K. SyRI: finding genomic rearrangements and local sequence differences from whole-genome assemblies. Genome Biology. 2019;20:277. https://doi.org/10.1186/s13059-019-1911-0

28. Armstrong J, Hickey G, Diekhans M, Fiddes IT, Novak AM, Deran A, et al. Progressive Cactus is a multiple-genome aligner for the thousand-genome era. Nature. 2020;587:246–51. https://doi.org/10.1038/s41586-020-2871-y
